# High-Fluence Multi-Energy Ion Irradiation for Testing of Materials

**DOI:** 10.3390/ma15186443

**Published:** 2022-09-16

**Authors:** Pavol Noga, Zoltán Száraz, Matej Kubiš, Jozef Dobrovodský, Filip Ferenčík, Róbert Riedlmajer, Vladimir Krsjak

**Affiliations:** 1 Slovak University of Technology in Bratislava, Faculty of Materials Science and Technology in Trnava, Advanced Technologies Research Institute, Jána Bottu 25, 91724 Trnava, Slovakia; 2 Slovak University of Technology in Bratislava, Faculty of Electrical Engineering and Information Technology, Institute of Nuclear and Physical Engineering, Ilkovičova 3, 81219 Bratislava, Slovakia

**Keywords:** high-energy ion irradiation, nuclear materials, transmutation helium

## Abstract

Structural materials of the new generation of nuclear reactors, fission as well as fusion, must often cope with high production rates of transmutation helium. Their testing hence requires either a powerful source of fast neutrons or a high-fluence ion-irradiation facility providing sufficient amounts of high-energy helium to investigate its effect on the material. Most ion irradiation studies, however, concentrate on basic effects such as defect evolution or bubble swelling in narrow near-surface regions modified by ion bombardment. Studies on bulk samples with a relatively thick implanted region, which would enable, for instance, micromechanical testing, are underrepresented. This gap might be filled by high-fluence multi-energy ion irradiations modifying several tens of micrometres of the investigated substrate. High-energy ion accelerators providing reasonable currents with energies of tens of MeV are rarely employed in such studies due to their scarcity or considerable beamtime costs. To contribute to this field, this article reports a unique single-beam He implantation experiment aimed at obtaining quasi-uniform displacement damage across >60 μm with the He/dpa ratio roughly one order of magnitude above the typical spallation neutron target irradiation conditions. Some technical aspects of this irradiation experiment, along with recent developments and upgrades at the 6 MV Tandetron accelerator of the Slovak university of technology in Bratislava, are presented.

## 1. Introduction

The growing demand for more energy with simultaneous efforts towards carbon-free energy production increases the importance of the nuclear energy sector. In the recent years there has been an interest to develop fourth-generation (GEN IV) fission reactors, small modular reactors, and fusion reactors. Compared with the current reactor conditions, materials in advanced nuclear systems need to withstand higher temperatures, more corrosive coolants, and prolonged high-energy neutron irradiation. While the operating temperature of commercial light water reactors does not exceed 350 °C, the six concepts of the future fission systems, proposed within the Generation IV international forum, will operate in a temperature range of 350–1000 °C. The foreseen end of lifetime damage levels are up to ~200 dpa [1,2]. In prototype fusion devices the damage of 150–200 dpa in the replaceable structures will be caused by the 14 MeV neutrons generated during D-T fusion reaction. The demonstration fusion power plant DEMO is expected to operate from 300 to 1000 °C [3].

Higher neutron flux and harder neutron spectra, together with increased temperatures, call for the development of new radiation-tolerant structural materials. For the deployment of the abovementioned new systems, it is crucial to understand how radiation degrades these materials and how various parameters affect their irradiation response. The materials development is a challenging, lengthy process as it needs to go through several steps and iterations, and the neutron irradiation to significant exposures takes a long time. Other limiting factors are the decreased availability of suitable materials test reactors and the very high cost of neutron irradiation. To shorten the 40–50-year process, materials development with a focus on high-fluence irradiation conditions uses the best alternative technique available to date: charged particle irradiation, at least until facilities such as the High Flux Accelerator-Driven Neutron Facility (HF-ADNeF) [4,5] or the much more powerful IFMIF-DONES facility [6] capable of producing a neutron spectrum very close to the conditions in fusion reactors and at high fluxes become operational. Yet, operation of the latter is planned no earlier than 2033 [7].

Ion irradiation is widely employed to investigate radiation-induced microstructural changes and the resultant material damage. Ion implantation enables much faster damage accumulation in comparison with nuclear reactors, and the dose corresponding to several years of neutron irradiation can be reached in a matter of hours or days and is therefore considered as the best available surrogate to neutron irradiation known to date. However, the penetration of the accelerated ions into the material is limited. The irradiation depth achieved during low energy proton or heavy ion irradiation (up to 100 MeV) is in the order of submicron to a few micrometres for common metals used in nuclear materials [8]. Therefore, the damage is confined into a thin near-surface region, in the order of μm, and the resulting displacement profile is graded. This makes it difficult to determine and evaluate the mechanical properties of ion-irradiated materials and limits the testing and investigation to the nanoscale level. Most ion irradiation facilities for materials research provide relatively low-energy protons or heavy ions (2–5 MeV), restricting the investigation of irradiation induced changes in mechanical properties under irradiation. Moreover, most such investigations limit themselves to a single implantation/irradiation step.

For materials development and qualification, engineering data such as strength, ductility, toughness, etc. are needed. To extract bulk properties, the range limitation in ion implantation experiments needs to be overcome. Increasing the irradiated layer thickness to 50–100 μm or beyond will make micro-mechanical testing [9] feasible.

Obtaining such “bulk” volumes of ion-beam modified material requires employing high-energy ion accelerators with powerful ion sources. Ion bombardment using light particles with energy > 10 MeV, however, leads to considerable amounts of gamma and neutron radiation during the beam-on time. This must be considered in the accelerator facility layout and resolved by sufficient radiation shielding.

One of the few setups dedicated especially to “thick layer” (in sense of the above) irradiation is located at MIT [8], based on a cyclotron providing 10–30 MeV proton irradiations and a full mechanical tensile test stage with 100–300 μm-thick samples (proton ranges at these energies ensure almost complete transmission through the foil).

Protons reasonably simulate ballistic effects when considering particle size and mass [10,11]. Nevertheless, transmutation products such as helium with a critical contribution to damage evolution [12] cannot be addressed in proton irradiation studies. Self-ion irradiation as a surrogate for neutron irradiation was proved as feasible in various studies [11,13,14,15,16,17] focused on void swelling, but has been almost exclusively limited to TEM characterisation of regions a few microns thick.

Most irradiation studies are conducted at fluences up to ~10^17^ at/cm^2^ [18] and in case of higher MeV energies these numbers are usually a few orders less. To be noted, however, these studies aimed at the investigation of basic ion-matter interaction and defect evolution. There are, however, few experiments with fluences surpassing that, such as the 5.42 × 10^19^ at/cm^2^ proton irradiation performed recently [19]. Yet, to study engineering-relevant properties, bulk properties, the whole volume is to be irradiated homogeneously to introduce a quasi-uniform damage in terms of dpa, as well as in the case of evaluating the effect of transmutation helium, a “box-profile” of He concentration.

The ion beam centre at ATRI MTF STU recently upgraded its ion source systems to serve high-fluence high energy ion irradiations comprising a high-current upgrade of the HVEE 358 Duoplasmatron ion source and the installation of a NEC TORVIS (Toroidal Volume Ion Source).

The aim is to perform multi-step ion-irradiation with different ion energies to achieve a nearly flat dpa-profile and almost constant irradiation hardening over the irradiated layer. The results of nanoindentation performed on such specimens will be easier to interpret as the substrate layer will not play a role in the results. Moreover, multi-step irradiation will increase the layer thickness compared with single step irradiation, approximately by a factor of 100. In this way, irradiation depths of ~70 µm can be achieved in steels, which allows applying micromechanical tests such as micropillar compression. This thickness of the radiation damage layer is not only sufficient for some micro-mechanical testing methods, but also for the application of conventional (radioisotope-based) positron sources in the techniques of positron annihilation spectroscopy (PAS), which is one of the important characterisation methods used in the post-irradiation examination (PIE).

## 2. Materials and Methods

### 2.1. Equipment

**Figure 1:**
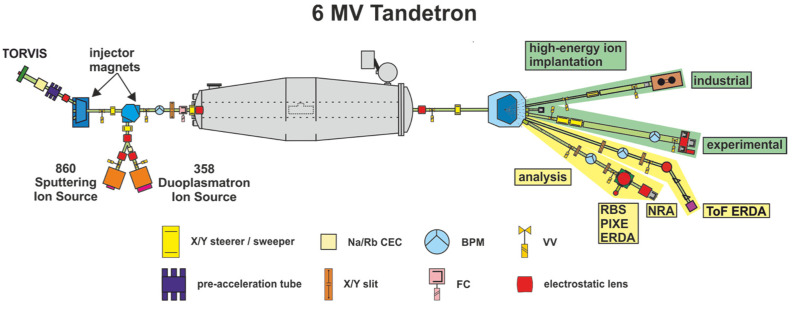
Schematic of the 6 MV Tandetron tandem accelerator setup.

The 6 MV tandem accelerator setup of the Ion beam laboratory at ATRI MTF STU [20] has recently undergone upgrades, increasing the beam currents for proton and helium ions as well as the provision of end stations for performing high-fluence irradiation experiments. The new setup, Figure 1, has an upgraded HVEE 358 Duoplasmatron ion source with modified extraction optics and a new Na Charge-exchange canal (CEC) designed to deliver He^−^ beam currents up to 8 µA. The second addition is a used NEC TORVIS [21] with an Rb-CEC designed to deliver He^−^ and proton beams up to 20 µA and 100 µA respectively. These ion sources are complemented by a HVEE 860 Cs sputtering ion source for heavy ion beams. The related vacuum system is completely oil-free with a base vacuum level better than 5.0 × 10^−7^ mbar.

**Figure 2:**
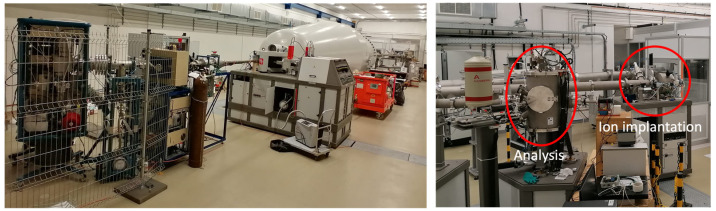
The 6 MV Tandetron accelerator with ion sources (**left**) and end-stations (**right**).

On the high-energy side, the system is equipped with end-stations for analysis and high energy ion implantation/irradiation, Figure 2. The analytical end-station is equipped with standard Rutherford Backscattering Spectrometry (RBS), Particle Induced X-ray Emmission (PIXE), and basic Elastic Recoil Detection Analysis (ERDA) for hydrogen and Nuclear Reaction Analysis (NRA). A detailed description of the analytical system can be found elsewhere [22]. The second analytical end-station, currently under procurement, will be equipped with a Time-of-Flight ERDA (ToF ERDA) spectrometer based on the Jyväskylä design [23] and will enable highly sensitive elemental composition analysis without the need for reference materials.

High-energy ion implantation/irradiation is served by two end-stations, a commercial semiconductor wafer handling system (client property), and another one for experimental purposes. The latter enables ion implantation/irradiation of substrates with sizes up to Ø100 mm at room temperature and sample cooling down to LN2 temperatures. Sample heating up to 1000 °C is possible with Ø40 mm sample holding space. The usual experimental setup for high-fluence experiments is a water-cooled Ø40 mm sample holder where the beam is rastered over an area of 36 cm^2^, which is given by the sample holding space and related current measurement system. With this setup, the ion currents abovementioned translate to a helium flux of 1.0 × 10^12^ at/cm^2^/s and proton flux up to 1.2 × 10^13^ at/cm^2^/s.

### 2.2. Multi Energy Sequential Irradiation Experiment Design

**Figure 3:**
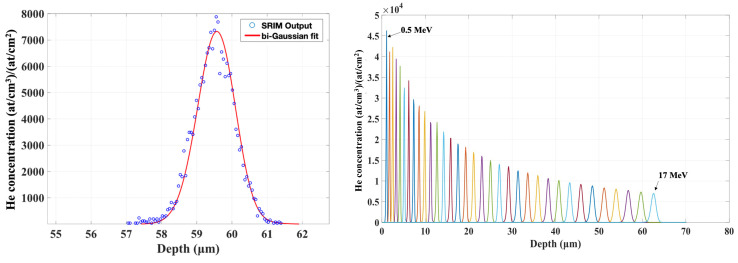
Fitting of individual concentration profiles (**left**) and the resulting profile-set (**right**).

Irradiation experiments aiming to investigate the effect of transmutation helium require a homogeneous He concentration distribution. We adopted a similar approach as that in ref. [24], wherein the authors “assembled” dopant box-profiles from a sequence of ion implantation steps with decreasing ion energies. First, the implantation depth profiles at individual ion energies were calculated using SRIM [25] and fitted using a suitable function. The ion ranges were Gaussian-like by nature and bi-Gaussian functions yielded the best fitting, Figure 3. Since the investigated materials within the ongoing research projects are mostly nuclear grade ferritic/martensitic steels, all SRIM calculations of range as well as displacement damage profiles used ^56^Fe as the target material. The number of energies to be employed is a parameter of choice and has to be chosen reasonably with respect to the system’s switching and re-tuning time constants.

The ion fluence at individual energies was calculated by solving the following minimisation problem:

(1) $$\underset{u_{1\ldots n}}{\min}||\sum_{i=1}^{n}C_{\mathrm{He}}{}_{i}\left(x\right)u_{i}-W\left(x\right)||\;\mathrm{subject}\;\mathrm{to}\;\sum_{i=1}^{n}u_{i}=\int W\left(x\right)dx,$$

where $C_{\mathrm{He}}{}_{i}\left(x\right)$ is the *i*-th helium concentration profile, W(x) is the desired concentration profile (in our case a “box-profile”), $u_{i}$ are ion fluences at individual energies, *n* is the number of implantation steps/energies. The resultant implantation profile becomes

(2) $$\hat{W}\left(x\right)=\overset{n}{\underset{i=1}{\sum }}C_{\mathrm{He}}{}_{i}\left(x\right){\hat{u}}_{i}$$

where ${\hat{u}}_{i}$ are calculated optimal fluences at individual energies.

In light of the above, we decided to assemble the final profile of 34 individual ion implantations starting at 17 MeV going down to 500 keV in 500 keV steps, Figure 3. Our experiment aimed at reaching minimum 1000 appm (atomic ppm) He concentration, which yields bubble sizes suitable for TEM observation, as our previous research showed [12]. This concentration corresponds to a fluence of 5.42 × 10^17^ at/cm^2^ and was limited by the achievable ion-beam current and acceptable duration, i.e., cost, of the experiment. The total irradiation time is on the order of a few hundreds of hours and was performed using the water-cooled sample holder kept at room temperature to avoid temperature effects. The resulting displacement damage across the irradiated region was calculated to 0.162 dpa, according to suggestions and recommendations published in ref. [26] using the NRT model [27]. It is important to note that the resulting He/dpa ratio is approximately 50× higher than the typical irradiation conditions of the spallation neutron targets (~100 appm He/dpa). Despite this, the helium concentration is almost two orders of magnitude higher than the expected helium production in fusion tokamaks; the planned microstructural characterisation and micromechanical testing can provide valuable experimental data to the material research for both fusion and spallation environments.

## 3. Results and Discussion

### 3.1. Upgraded Equipment

The upgraded HVEE 358 duoplasmatron ion source routinely operates with a 3–7 µA He^−^ injection current. Ion transport efficiency through the accelerator is still to be improved, as it is roughly about 50% just due to losses in the Ar stripper channel. The NEC TORVIS system was tested with hydrogen as well as helium, where we achieved stable proton currents around 30 µA, and 4 µA for helium. Much more is expected; however, these values were achieved during the first runs after the revival of the TORVIS and further tuning will increase ion yield.

### 3.2. High-Fluence Helium Irradiation

**Figure 4:**
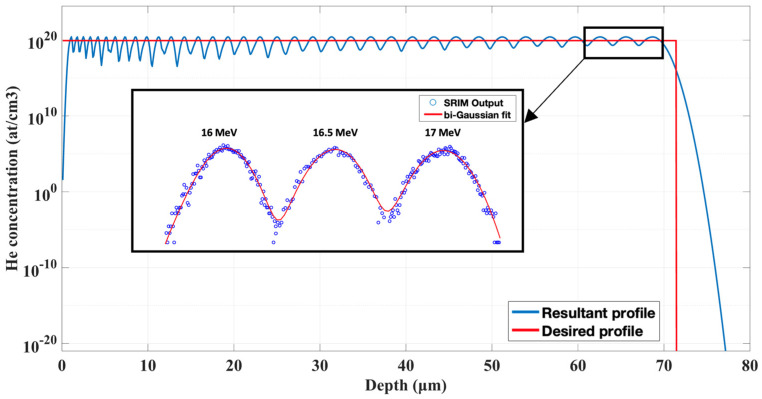
The resulting helium concentration profile assembled of 34 implantation steps.

The multi-energy ion implantation experiment yields a 65 µm-thick irradiated layer with 1000 appm implanted helium, approximately homogeneously distributed in the layer, Figure 4. This makes micromechanical testing by micropillar compression as well as microcantilever bending possible in reasonable pillar and cantilever sizes to extract engineering-relevant data.

**Figure 5:**
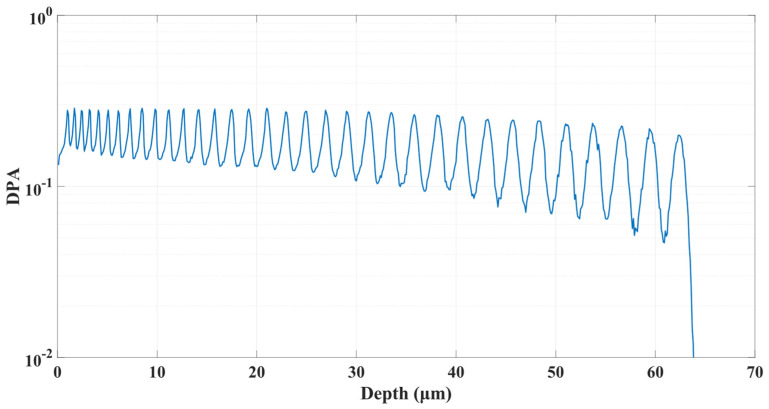
Resultant damage profile after 34 implantation steps.

Accumulated irradiation damage was 0.162 dpa and was distributed in accordance with the irradiation profile, Figure 5. In comparison with using degrader foils [28], this approach enables better control of the helium concentration as well as damage profiles.

When considering irradiation fluxes, one has to keep in mind that aside from sample heating, which in our case was mitigated by a water-cooled copper/aluminium sample holder, 17 MeV He irradiation of Fe–Cr-based alloys produces a significant amount of neutron and gamma radiation. Our measurements indicated neutron dose rates up to 4.5 mSv/h/μA and gamma dose rates up to 0.5 mSv/h/μA at 1 m distance from the irradiation spot. The beam was He^2+^, and hence 1 μA represents 3.12 × 10^12^ alpha particles per second. After the test phase, the chamber was additionally shielded with 5 cm lead shielding to protect the equipment and electronics present in the laboratory. Nevertheless, the laboratory is shielded by 1.5 m thick high-density concrete (3.8 g/cm^3^) shielding, which attenuates the radiation down to background levels at the outer walls even at maximum beam currents [29].

## 4. Conclusions

The ATRI MTF STU ion beam laboratory upgraded its 6 MV tandem accelerator setup. High-current ion sources, the upgraded HVEE 358 duoplasmatron, and the NEC TORVIS increased current output especially for helium, enabling unique irradiation studies of radiation effects in fusion or spallation structural materials. The first tests were performed, and after further tuning the system is expected to deliver 10 µA He and 50–100 µA proton beams in the experimental chamber. Maximum sample size with heating up to 1000 °C or water cooling is Ø40 mm, otherwise up to Ø100 mm. The laboratory operates in open-access mode. The planned multi-energy high-fluence irradiation experiment will provide a 65 µm-thick approximately homogeneously irradiated layer in steels, enabling micromechanical testing and the evaluation of engineering-relevant properties of the irradiated materials.

## References

[1] DOENE (2002). A Technology Roadmap for Generation IV Nuclear Energy Systems.

[2] Stanculescu A. GIF R&D Outlook for Generation IV Nuclear Energy Systems: 2018 Update. Proceedings of the Generation IV International Forum.

[3] Zinkle S.J., Snead L.L. (2014). Designing Radiation Resistance in Materials for Fusion Energy. Annu. Rev. Mater. Res..

[4] Wheldon C. Applications in Nuclear Physics and Nuclear Industry. Proceedings of the PSD12: The 12th International Conference on Position Sensitive Detectors.

[5] University of Birmingham High Flux Accelerator-Driven Neutron Facility. https://www.birmingham.ac.uk/research/activity/nuclear/about-us/facilities/high-flux-neutron-facility.aspx.

[6] Bernardi D., Arbeiter F., Cappelli M., Fischer U., García A., Heidinger R., Krolas W., Martín-Fuertes F., Miccichè G., Muñoz A. (2019). Towards the EU fusion-oriented neutron source: The preliminary engineering design of IFMIF-DONES. Fusion Eng. Des..

[7] ESFRI (2021). ESFRI Roadmap 2021 Strategy Report on Research Infrastructures.

[8] Jepeal S.J., Danagoulian A., Kesler L.A., Korsun D.A., Lee H.Y., Schwartz N., Sorbom B.N., Velez Lopez E., Hartwig Z.S. (2021). An accelerator facility for intermediate energy proton irradiation and testing of nuclear materials. Nucl. Instrum. Meth. Phys. Res. B.

[9] Hosemann P. (2018). Small-scale mechanical testing on nuclear materials: Bridging the experimental length-scale gap. Scripta Mater..

[10] Rayaprolu R., Möller S., Linsmeier C., Spellerberg S. (2016). Simulation of neutron irradiation damage in tungsten using higher energy protons. Nucl. Mater. Energy.

[11] Was G.S. (2015). Challenges to the use of ion irradiation for emulating reactor irradiation. J. Mater. Res..

[12] Krsjak V., Shen T., Degmova J., Sojak S., Korpas E., Noga P., Egger W., Li B., Slugen V., Garner F.A. (2022). On the helium bubble swelling in nano-oxide dispersion-strengthened steels. J. Mater. Sci. Technol..

[13] Garner F.A. (1983). Impact of the injected interstitial on the correlation of charged particle and neutron-induced radiation damage. J. Nucl. Mater..

[14] Was G.S., Jiao Z., Getto E., Sun K., Monterros A.M., Maloy S.A., Anderoglu O., Sencer B.H., Hackett M. (2014). Emulation of reactor irradiation damage using ion beams. Scr. Mater..

[15] Gigax J.G., Kim H., Chen T., Garner F.A., Shao L. (2017). Radiation instability of equal channel angular extruded T91 at ultra-high damage levels. Acta Mater..

[16] Shao L., Wei C.-C., Gigax J., Aitkaliyeva A., Chen D., Sencer B.H., Garner F.A. (2014). Effect of defect imbalance on void swelling distributions produced in pure iron irradiated with 3.5 MeV self-ions. J. Nucl. Mater..

[17] Kumar N.A.P.K., Li C., Leonard K.J., Bei H., Zinkle S.J. (2016). Microstructural stability and mechanical behavior of FeNiMnCr high entropy alloy under ion irradiation. Acta Mater..

[18] Krsjak V., Degmova J., Noga P., Petriska M., Sojak S., Saro M., Neuhold I., Slugen V. (2021). Application of positron annihilation spectroscopy in accelerator-based irradiation experiments. Materials.

[19] Shiau C.-H., Sun C., McMurtrey M., O’Brien R., Garner F.A., Shao L. (2022). Orientation-selected micro-pillar compression of additively manufactured 316L stainless steels: Comparison of as-manufactured, annealed, and proton-irradiated variants. J. Nucl. Mater..

[20] Noga P., Dobrovodský J., Vaňa D., Beňo M., Závacká A., Muška M., Halgaš R., Minárik S., Riedlmajer R. (2017). A new ion-beam laboratory for materials research at the Slovak University of Technolog. Nucl. Insturm. Meth. Phys. Res. B.

[21] Hauser T.M., Daniel R.E., Norton G.A., Schroeder J.B. (2006). High current He^-^ injector for tandem accelerators. Nucl. Instrum. Meth. Phys. Res. B.

[22] Dobrovodský J., Beňo M., Vaňa D., Bezák P., Noga P. (2019). The first year operation experience with Ion Beam Analysis at the new STU Ion Beam Laboratory. Nucl. Insturm. Meth. Phys. Res. B.

[23] Laitinen M., Rossi M., Julin J., Sajavaara T. (2014). Time-of-flight—Energy spectrometer for elemental depth profiling—Jyväskylä design. Nucl. Insturm. Meth. Phys. Res. B.

[24] Wu H., Böttger R., Couffignal F., Gutzmer J., Krause J., Munnik F., Renno A.D., Hübner R., Wiedenbeck M., Ziegenrücker R. (2019). ‘Box-Profile’ Ion Implants as Geochemical Reference Materials for Electron Probe Microanalysis and Secondary Ion Mass Spectrometry. Geostand. Geoanal. Res..

[25] Ziegler J.F., Ziegler M.D., Biersack J.P. (2010). SRIM—The stopping and range of ions in matter. Nucl. Insturm. Meth. Phys. Res. B.

[26] Stoller R.E., Toloczko M.B., Was G.S., Certain A.G., Dwaraknath S., Garner F.A. (2013). On the use of SRIM for computing radiation damage exposure. Nucl. Insturm. Meth. Phys. Res. B.

[27] Norgett M.J., Robinson M.T., Torrens I.M. (1975). A proposed method of calculating displacement dose rates. Nucl. Eng. Des..

[28] Brimbal D., Meslin E., Henry J., Décamps B., Barbu A. (2013). He and Cr effects on radiation damage formation in ion-irradiated pure iron and Fe-5.40 wt.% Cr: A transmission electron microscopy study. Acta Mater..

[29] (2015). Radiation Safety Report on Radiation Shielding Test of CAMBO Ion Beam Centre Building.

